# Novel in-office peroxide-free tooth-whitening gels: bleaching effectiveness, enamel surface alterations, and cell viability

**DOI:** 10.1038/s41598-020-66733-z

**Published:** 2020-06-22

**Authors:** Juliana Silva Ribeiro, Andressa da Silva Barboza, Carlos Enrique Cuevas-Suárez, Adriana Fernandes da Silva, Evandro Piva, Rafael Guerra Lund

**Affiliations:** 1 Graduate Program in Dentistry, Pelotas Dental School, UFPel-Federal University of Pelotas, Gonçalves Chaves 457, Pelotas, 96015-560 Rio Grande do Sul Brazil; 2 Department of Cariology, Restorative Sciences and Endodontics, University of Michigan School of Dentistry, 3570 GreenBrier Boulevard 380 A, 48105 Ann Arbor, MI USA; 3 Dental Materials Laboratory, Academic Area of Dentistry, Autonomous University of Hidalgo State, Circuito Ex hacienda la Concepción S/N, San Agustín Tlaxiaca, 42060 Hidalgo Mexico; 4 Department of Restorative Dentistry, Pelotas Dental School, UFPel-Federal University of Pelotas, Gonçalves Chaves 457, Pelotas, 96015-560 Rio Grande do Sul Brazil

**Keywords:** Dental materials, Restorative dentistry

## Abstract

To evaluate the bleaching ability, the effect on enamel surface and cytotoxicity of novel tooth-whitening formulations containing papain, ficin, or bromelain. Forty bovine dental discs (6 cm ×4 cm) were pigmentated and randomly allocated into the following groups (n = 10): Group 1, 20 wt% carbamide peroxide (control); group 2, 1% papain-based whitening; group 3, 1% ficin-based whitening; and group 4, 1% bromelain-based whitening. The whitening gels were prepared and applied on the enamel three times per day once a week, for 4 weeks. Color measurement was obtained by CIEDE2000. Enamel Knoop microhardness and roughness were evaluated. The WST-1 assay was used to evaluate the cell viability of mouse fibroblast cells (L929). Data were statistically analyzed by one-way analysis of variance (ANOVA) and Student Newman Keuls’s post hoc test at α = 0.05 significance level. Bromelain, ficin-based, and carbamide peroxide bleaching gels showed a similar color change (p < 0.001). Higher enamel hardness decrease and higher enamel roughness were caused by the carbamide peroxide (p < 0.05). The experimental whitening gels did not affect cell viability. Tooth bleaching gels containing bromelain, papain, or ficin have substantial clinical potential to be used in the development of peroxide-free tooth whitening gels.

## Introduction

Tooth whitening has become one of the most performed and demanded cosmetic dental procedures. It is a non-invasive treatment that improves the appearance of a patient smile, positively affecting the quality of life^1,2^. The two whitening techniques traditionally used are the at-home and the in-office whitening techniques. Both techniques involve the use of different concentrations of carbamide or hydrogen peroxide as active agents^3^. Concentrations of such active agents commonly used for in-office whitening therapy vary from 20%, 35% or even 38%^4^.

Despite that, the efficiency of whitening systems is consolidated in the literature and dental practice, incorrect application in excessive quantities of time can cause side effects^5^. The most common drawback caused by the use of high hydrogen peroxide concentrations is tooth sensitivity^6^. A study demonstrated that 85% of patients treated using an in-office bleaching technique report tooth sensitivity^7,8^. It was hypothesized that tooth sensitivity after dental bleaching is related to the degradation of the hydrogen peroxide into reactive oxygen species (ROS) which can diffuse to the pulp chamber through the dentinal tubules, inducing the release of inflammatory mediators, like IL-1β and RANK-L^9,10^. Although the use of analgesics, anti-inflammatories, antioxidants, corticosteroids, and opioid drugs are used as a way to minimize this side-effect, the use of oral medications is not able to reduce the risk of tooth sensitivity^11^.

An alternative to reduce or solve this problem is the use of natural compounds. Given their natural origin, these types of products have a certain guarantee on their non-toxic biological behavior, resulting in little or no harmful effects^12^. For example, a diet rich in some types of fruits or vegetables, which contains large amounts of organic acids, seems to preserve or improve the color of the teeth^13^. Thus, we hypothesized that the development of new whitening agents based on natural products could provide similar aesthetic results and minor side effects^12^. Up to now, it seems that despite some papers relate the use of natural compounds as dental bleaching agents, these were not more effective than the traditional techniques^14^.

Considering the deleterious effects that peroxide bleaching agents bring to the dental structure, some alternatives have already been studied^15–19^. Among them, the cysteine protease enzymes, such as papain and bromelain have been described as active agents with bleaching potential^16,18,19^. The main characteristic of this type of enzymes is the capability to improve the hydrogen peroxide-dependent oxidation-reduction and reduce the toxicity of electron donors compounds, such as peroxides and some aromatic compounds^20^. Papain, bromelain, and ficin are widely used because of their properties, such as anti-inflammatory, antithrombotic and fibrinolytic activity, anticancer activity, and immunomodulatory effects^21–23^. In addition, ficin is a cysteine from the protease group which is widely distributed among living organisms^24^. Due to their enzymatic feature, these enzimes could be considered as an active agents with a whitening effect.

To the extent of our knowledge, the stain removal ability of ficin cysteine protease has not been studied; thus, the objective of this work was to evaluate *in vitro* the bleaching potential of non-peroxide whitening gels containing bromelain, papain, or ficin as active agents. The null hypothesis tested was that different whitening gels formulated will not affect the color change, microhardness and roughness of the enamel-dentin discs, and the cell viability of mouse fibroblast cells (L929).

## Materials and methods

### Preparation of the peroxide-free tooth-whitening gels

Three experimental gels containing papain, bromelain, or ficin were prepared using the reagents shown in Table 1. The formulation was prepared as a viscous gel at room temperature. Carbopol® which was used as a thickener was incorporated into propylene glycol and mixed until a homogeneous gel was obtained. Water-soluble components such as potassium oxalate, sodium fluoride, and preservatives were previously solubilized in ultrapure water and added to the gel until its homogenization. Then, the enzymes were incorporated until the formation of a homogeneous and transparent gel resulting in 1% wt of bromelain-, ficin, or papain-based gel, respectively. To utilize the enzymes in their maximal activity, the pH of the gels was adjusted to the optimum pH for proteolytic activity by adding a sodium hydroxide solution. pH was measured using a previously calibrated digital pH meter (Quimis®, Diadema - SP - Brazil).

**Table 1:** Components of gel formulation used as vehicle.

Ingredient	Experimental Groups			
	Bromelain-based gel	Ficin-based gel	Papain-based gel	Negative Control
Bromelain (Sigma-Aldrich St. Louis, MO, USA)	1%	—	—	—
Ficin (Sigma-Aldrich)	—	1%	—	—
Papain (Sigma-Aldrich)	—	—	1%	—
Potassium oxalate (Sigma-Aldrich)	0.3%	0.3%	0.3%	0.3%
Sodium Fluoride (Sigma-Aldrich)	0.2%	0.2%	0.2%	0.2%
Propylene glycol (Sigma-Aldrich)	35%	35%	35%	35%
Carbopol® (Lubrizol, Wickliffe, Ohio, USA)	1%	1%	1%	1%
Sodium benzoate (Sigma-Aldrich)	0.2%	0.2%	0.2%	0.2%
Ultrapure water	Qsp	Qsp	Qsp	Qsp
pH*	7.2–7.5	7–8	6.5	—

*Adjusted by adding a sodium hydroxide solution.

### Specimens preparation

Fifty bovine teeth were donated by the city’s abattoir (Famile abattoir, Pelotas, RS. Brazil) and stored in 0.1% thymol solution for 1 week. After cleaning with distilled water, the root was sectioned, and their crowns were embedded in high-fusion impression compound, allowing the buccal enamel surface to be exposed. Standard enamel-dentin discs 6 cm in diameter and 4 cm in thickness were cut from the buccal enamel surface using a water-cooled trephine drill. The discs were wet-polished with 600 and 1200 grit silicon carbide papers. After polishing, enamel was etched using 37% phosphoric acid (Total-etch; Ivoclar-Vivadent, Amherst, NY, USA) for 60 seconds and rinsed with water for 30 seconds^3^. Then, the prepared specimens were stored in a coffee solution for 1 week at 37 °C. The coffee solution was prepared by boiling 12 g of coffee (Melitta, Avaré, SP, Brazil) in 200 ml of distilled water for 5 min followed by filtering. The storage media was replaced with a fresh solution daily^3^. After pigmentation, the specimens were washed with distilled water and left in a culture plate (24-well plates) filled with distilled water to maintain the moisture of the specimens. The 24-well plates were kept at 37 °C in an incubator for 24 h.

### Stain removal procedures

After the staining process, initial measurements of color, hardness loss, and surface roughness were performed in all the groups. Then, all the specimens were randomly allocated to the following groups (n = 10): carbamide peroxide (control); bromelain-based whitening gel; papain-based whitening gel; and ficin-based whitening gel. A negative control (distilled water) was also analyzed. Considering that the experimental bleaching agents do not have any standardized application protocol, all the whitening gels were applied following a protocol based on a previous study^16^. The gels were placed in the enamel and left undisturbed for 15 min. During the application, specimens were kept in a humid environment by placing them over a moistened gauze. The gel was then washed using a gauze soaked in water. This procedure was repeated four times, simulating four clinical applications with a 1-week interval. Between each application, the specimens were kept in artificial saliva^16^.

### Color change

To evaluate the color change of the specimens, the CIELAB color parameters of enamel specimens were measured by means of sphere spectrophotometer (SP60, X-Rite^©^; MI, USA). The specimens were evaluated against a white background before (baseline color) and after the bleaching protocols. The reference values used were those obtained after coffee pigmentation. The color differences (ΔE) with the CIEDE 2000 formula were calculated^25^.

The color change was also calculated as a function of the whiteness index (WI_D_). The WI_D_ of the specimens after the pigmentation process (WI_D1_) and after the stain removal protocol (WI_D2_) was calculated using the following formula^26^:

$$W{I}_{D}=0.511L-2.324a-1.100b$$

where WI_D_ is the whitening index, 0.511 is the constant of lightness, 2.32 is the constant for the coordinates from green to red, and 1.100 is the constant for the coordinates from blue to yellow.

The difference in the WI_D_ between the two measurements was calculated in percentage using the following formula:

$$ \% {{\rm{DWI}}}_{{\rm{D}}}=({{\rm{WI}}}_{{\rm{D}}2}-{{\rm{WI}}}_{{\rm{D}}1})\times 100/{{\rm{WI}}}_{{\rm{D}}1}.$$

### Hardness loss

Before exposure to whitening agents, the enamel surface Knoop microhardness (MH1) was obtained using a microdurometer (FM-700, Future-Tech^®^ Corporation; Kanagawa, Japan) using a 25 g load applied for 10 s. Three indentations, on the enamel surface, at 25, 50, and 100 μm from the margins of each specimen were performed parallel to the surface interface of the dental enamel. For each specimen, the results obtained from these three indentations were averaged and used for statistical purposes (n = 10). After the bleaching process, the microhardness measurement was repeated under the same conditions (MH2). The surface hardness loss (% SS) was calculated in percentage using the following formula:

$$ \% {\rm{SS}}=({\rm{MH}}2-{\rm{MH}}1)\times 100/{\rm{MH}}1.$$

### Surface roughness

The enamel roughness of each specimen was evaluated by means of roughness portable measuring instrument (Surftest SJ-301, Mitotoyo; Kanagawa, Japan) using the following parameters: cut-off length of 1.25 mm (lc) and 0.25 mm (ls), and cutting speed of 0.5 mm/s. For each specimen, the results obtained from five measurements were averaged and the mean roughness, in Ra, was used for statistical purposes (n = 10). One calibration block was used every six specimens to verify the performance of the profilometer. Roughness was evaluated before (Ra1) and after (Ra2) the bleaching protocols. The difference of surface roughness was calculated (D Ra %) using the following formula:

$$ \% {\rm{D}}\,{\rm{Ra}}=({\rm{Ra}}2-{\rm{Ra}}1)\times 100/{\rm{Ra}}1.$$

### Cell viability

L929 mouse fibroblasts were cultured in DMEM (Dulbecco´s Modified Eagle´s medium) cell culture medium supplemented with 10% fetal bovine serum, 10% L-glutamine, and 100 units/mL penicillin/streptomycin. Cells were incubated at a density of 2 × 10^4^ cells in 96-well plates at 37 °C for 24 h in an air atmosphere containing 95% air and 5% CO_2_.

Whitening gels samples (~50 µg) from each group (n = 6) were placed in 24-well plates containing 1 mL of DMEM (pH 7.2). After 45 min of incubation at 37 °C, 200 μL of the conditioned culture medium was transferred to the plates containing the pre-cultured cells. The plate was then incubated at 37 °C in an air atmosphere containing 95% air and 5% CO_2_ for a period of 24 h. After this period, the culture medium was replaced by a WST-1 solution (Roche Life Science; Penzberg, Germany) to assess cell metabolic function. The results were read in a microplate reader (SpectraMax M5; Molecular Devices, Sunnyvale, USA) with a wavelength of 450 nm. The absorbance of untreated cells was used as the control.

### Statistical analysis

The results of color change, microhardness, surface roughness, and cell viability were analyzed using IBM^®^ SPSS^®^ Statistics software (v 20.0; IBM^®^, USA). One-way analysis of variance followed by Student Newman Keuls’s post-hoc test were conducted to determinate statistically significant differences among the groups. Data from cell viability was analyzed through the one-way ANOVA on ranks and Tukey’s post-hoc test. The significance level was chosen at α = 5%.

## Results

**Figure 1:**
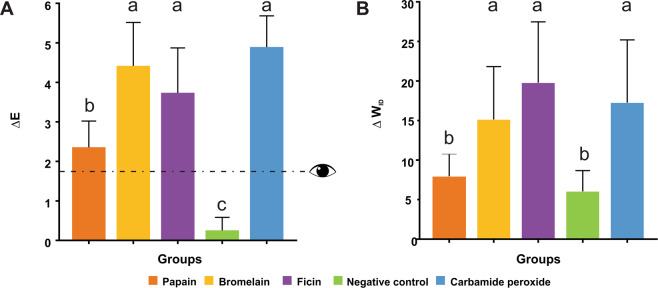
(**A**) Mean values and standard deviations of color difference (ΔE) after the application of bleaching gels. (**B**) Mean values and standard deviations of ΔW_ID_. Different lowercase letters indicate differences between bleaching gels (p < 0.05). The value indicated by the dotted line at ΔE = 1.8 corresponds to 50% visual perceptibility under clinical conditions and is used here as a reference for visually detectable color changes.

After the bleaching processes, all the gels promoted a greater color change than the negative control (Fig. 1; p < 0.001). Bromelain and ficin-based bleaching gels resulted in a similar color change than the carbamide peroxide gel (p < 0.05). Figure 1B shows the whiteness index alteration (ΔW_ID_) of the materials evaluated. Bromelain, Ficin, and carbamide peroxide gels achieved the highest increase in the W_ID_.

Tables 2 and 3, shows means and standard deviation of the hardness and roughness variation after the bleaching procedures. The carbamide peroxide showed the highest hardness loss (p < 0.001), followed by the bromelain group. After the bleaching procedure, the highest roughness increase was observed in the carbamide peroxide group (p < 0.001).

**Table 2:** Initial, final and variation of Knoop hardness (%) after bleaching procedures (Mean ± SD).

Group	Knoop Hardness (KHN)		
	Initial*	Final*	Δ KHN (%)^¶^
Carbamide peroxide	^A^ 121.44 ± 30.36^a^ (95% CI 103 to 140)	^B^ 99.48 ± 27.20^b^ (95% CI 82.6 to 116)	−18.72 ± 4.21^d^
Bromelain	^A^ 144.13 ± 44.19^a^ (95% CI 117 to 172)	^B^ 140.05 ± 43.86^ab^ (95% CI 113 to 167)	−2.92 ± 1.5^c^
Ficin	^A^ 145.19 ± 20.80^a^ (95% CI 132 to 158)	^B^ 153.96 ± 23.54^a^ (95% CI 139 to 169)	5.92 ± 2.5^a^
Papain	^A^ 136.95 ± 18.51^a^ (95% CI 125 to 148)	^B^ 162.31 ± 21.46^a^ (95% CI 149 to 176)	18.58 ± 3.4^b^
Negative control	^A^ 143.55 ± 41.33^a^ (95% CI 118 to 169)	^B^ 146.75 ± 41.65^a^ (95% CI 121 to 173)	2.51 ± 2.2^a^

*Data analyzed through a Two-Way Repeated Measures ANOVA test. Different uppercase letters indicate statistically significant differences in the same row (p < 0.05). Different lowercase letters indicate statistically significant differences in the same column (p < 0.05). ^¶^Data analyzed through a One-Way ANOVA test. Different lowercase letters indicate statistically significant differences (p < 0.05).

**Table 3:** Initial, final and variation of Mean Roughness (%) after bleaching procedures (Mean ± SD).

Group	Mean Roughness (Ra)		
	Initial*	Final*	Δ Ra (%)^¶^
Carbamide peroxide	^A^ 0.50 ± 0.06^a^ (95% CI 0.463 to 0.537)	^B^ 1.17 ± 0.09^a^ (95% CI 1.11 to 1.23)	131.69 ± 11.3^c^
Bromelain	^A^ 0.51 ± 0.16^a^ (95% CI 0.411 to 0.609)	^B^ 0.88 ± 0.22^b^ (95% CI 0.744 to 1.02)	96.86 ± 9.8^bc^
Ficin	^A^ 0.47 ± 0.08^a^ (95% CI 0.42 to 0.52)	^B^ 0.80 ± 0.08^b^ (95% CI 0.75 to 0.85)	71.39 ± 14.9^bc^
Papain	^A^ 0.44 ± 0.02^a^ (95% CI 0.428 to 0.452)	^B^ 0.52 ± 0.04^c^ (95% CI 0.495 to 0.545)	17.21 ± 4.8^ab^
Negative control	^A^ 0.53 ± 0.14^a^ (95% CI 0.443 to 0.617)	^A^ 0.60 ± 0.07^c^ (95% CI 0.557 to 0.643)	2.55 ± 1.6^a^

*Data analyzed through a Two-Way Repeated Measures ANOVA test. Different uppercase letters indicate statistically significant differences in the same row (p < 0.05). Different lowercase letters indicate statistically significant differences in the same column (p < 0.05). ^¶^Data analyzed through a One-Way ANOVA test. Different lowercase letters indicate statistically significant differences (p < 0.05).

**Figure 2:**
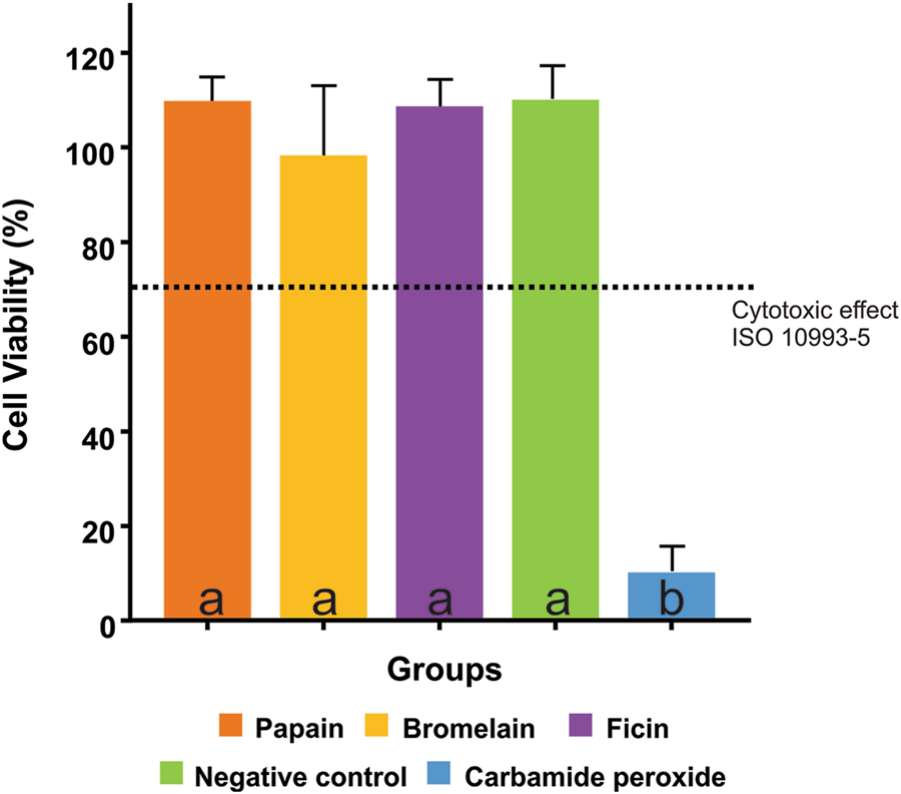
Results of cell viability assay taking into account the different groups. Different lowercase letters represent statistically significant differences between groups (p < 0.05). The value indicated by the dotted line at 70% corresponds to the minimum value of cell viability established by the standard to consider a material as non-cytotoxic.

The percentage of cell viability of fibroblast cells after incubation with eluate from different bleaching agents is shown in Fig. 2. Untreated group values were considered as 100% of cell viability. It was observed that bleaching gels based on natural enzymes, presented cell viability values near to 100%. Only carbamide peroxide gel promoted a cytotoxic effect against fibroblast cells (<70%).

## Discussion

This work evaluated the action of natural enzymes (ficin, bromelain, and papain) as non-peroxides dental whitening agents and its effect on the dental enamel surface and in cell viability. Considering the results obtained, the null hypothesis tested was partially rejected, since the ficin and bromelain-based gels promoted a greater color shift than the papain-based gel. Moreover, the enzyme-based gels presented less damage in the enamel surface and more cell viability than carbamide peroxide gel.

According to Fig. 1, all the experimental bleaching gels were effective in removing stains from the enamel surface, promoting a color change above the human perceptibility threshold. The color change caused by bromelain, ficin, and carbamide peroxide was greater than the human perceptibility threshold (0.8–1.8)^27^. The bromelain and ficin whitening gels showed similar color variation when compared to carbamide peroxide whitening gel. Some studies have reported that the stain removal effect of whitening agents could be caused by the rupture and removal of the stain-forming substances attached to the enamel surface^18^. Any molecule or stain attached to the tooth surface reduces light reflection, and consequently, the color of the tooth is altered^28^. As the enzymes used in this study are cysteine proteases, they can breakdown such macromolecules into smaller fractions, increasing the lightness of tooth, and therefore, its whiteness. This bleaching effect, similar to that observed by carbamide peroxide, is highly desired since tooth bleaching is associated with positive and stable impacts on aesthetic perception and psychosocial factors^29,30^.

As far as we know, there is only another study that has evaluated papain and bromelain as dental whitening agents; however, they showed a lower stain removal effect when compared to the carbamide peroxide^16^. This result could be explained because, in that study, the enzymes used were obtained by soy proteases, different from the present study where the enzymes were extracted from the plant itself, ensuring high purity. Additionally, in this study, the pH of the whitening gels was adjusted to ensure the optimum media for maximal enzyme activity^31^.

Another similar study^17^ tested the sweet potato (Ipomoea batatas L.) extract as a tooth-whitening agent. In such a study, sweet potato extract was used as an additive into a hydrogen peroxide-based gel and showed that the addition of such extract maintained the whitening potential of the hydrogen peroxide system, while the deleterious alterations in enamel morphology caused by the use of hydrogen peroxide alone were reduced. In the present study, all the experimental groups showed less enamel damage than the carbamide peroxide tooth-whitening gel (Tables 2 and 3). The carbamide peroxide caused the biggest hardness loss and the highest roughness increase. During the bleaching process, the peroxide decomposes easily when it reacts with compatible substances, resulting in the release of free radicals. When the tooth saturation point is reached, during decomposition, hydrogen peroxide reacts with the proteins and lipids from the tooth, removing them. This process results in the dissolution of the inorganic components of the enamel by penetrating into its intra and interprismatic areas^32^, making the surface of the enamel rougher and softener. This characteristic should be taken in the account since a relationship between a decrease in the hardness enamel with high susceptibility to caries disease has been documented^33^. In addition to this, such defects in the surface might interfere with the adhesive properties of restorative materials and should be taken in count in the planning phase of restorative treatment^34^.

With regards to the roughness surface, carbamide peroxide bleaching gel showed the highest increase after the bleaching process. It has been reported that this increase in the surface roughness increases the adhesion of Streptococcus mutans to enamel^35^ and could influence the formation of supra- and sub-gingival plaque^36^, with possible undesirable effects like the formation of carious decay. Also, the alteration of enamel roughness has been associated with enamel color change, which could impair the effectivity of bleaching procedures^37^. In this sense, the use of alternative bleaching agents, like papain, could prevent these undesirable effects.

Surprisingly, the papain and ficin whitening gels behaved as a protection factor increasing the hardness of the enamel surface when used with a remineralizing solution, the artificial saliva. The samples were maintained in artificial saliva during the experiments. This outcome could be due to the absence of free radicals release as it happens when the peroxides based gels are used; therefore, the oxidative reactions, which are the main mechanisms responsible for the toxicity and structural and biochemical damages on the dental hard and soft pulp tissues of peroxide-containing compounds^38^, do not occur. In an *in vitro* study^39^, an experimental natural bleaching gel led to a reduction in the color of dental composites without causing any alterations in surface morphology.

To perform the biocompatibility test, the cell viability was assessed after 45 min of exposure to simulate the dental clinical time exposure to bleaching gels. According to ISO 10993^40^, if a material has cell viability values below 70%, it is considered cytotoxic. Therefore, the experimental bleaching agents did not show any cytotoxic effect against mouse fibroblasts, while the peroxide carbamide showed values inferior to 30% showing cytotoxicity effect. We hypothesized that our results are in agreement with the literature once these enzymes have been widely used as having wound healing and anti-inflammatory on-cytotoxic biological behavior^22^.

Considering that these enzymes are widely used because of its anti-inflammatory properties^23^, the experimental bleaching gels formulated in this study could be able to reduce the tooth sensitivity, since ROS are not produced during the dental bleaching process. Considering the absence of ROS and the negative impact that this reactive species has on the performance of adhesive systems, the use of non-peroxide bleaching agents is desirable^41^.

Despite the adequate performance observed for the non-peroxide bleaching agents used, further research should be performed to evaluate the effects of these experimental bleaching agents in other cell lines, like dental pulp fibroblasts, stem cells, or gingival epithelial cells. Also, despite the good bleaching effects of bromelain, papain, and ficin observed, this cannot be extrapolated to a clinical scenario, and further *in-vivo* studies are needed to evaluate their performance.

## Conclusions

The non-peroxide bromelain and ficin gels were effective in dental bleaching, being similar to the carbamide peroxide-based gel. Moreover, the use of bromelain and ficin-based tooth-whitening gels resulted in less enamel damage than carbamide peroxide. These enzymes showed promising results, representing significant clinical potential as active ingredients of peroxide-free whitening products.
